# Sound source localization by *Ormia ochracea* inspired low–noise piezoelectric MEMS directional microphone

**DOI:** 10.1038/s41598-020-66489-6

**Published:** 2020-06-12

**Authors:** Ashiqur Rahaman, Byungki Kim

**Affiliations:** School of Mechatronics Engineering, Korea University of Technology and Education, Cheonan, 31253 Republic of Korea

**Keywords:** Electrical and electronic engineering, Mechanical engineering

## Abstract

The single-tone sound source localization (SSL) by majority of fly *Ormia ochracea*’s ears–inspired directional microphones leaves a limited choice when an application like hearing aid (HA) demands broadband SSL. Here, a piezoelectric MEMS directional microphone using a modified mechanical model of fly’s ear has been presented with primary focus to achieve SSL in most sensitive audio bands to mitigate the constraints of traditional SSL works. In the modified model, two optimized rectangular diaphragms have been pivoted by four optimized torsional beams; while the backside of the whole structure has been etched. As a result, the SSL relative to angular rotation of the incoming sound depicts the cosine dependency as an ideal pressure–gradient sensor. At the same time, the mechanical coupling leads the magnitude difference between two diaphragms which has been accounted as SSL in frequency domain. The idea behind this work has been analytical simulated first, and with the convincing mechanical results, the designed bio–inspired directional microphone (BDM) has been fabricated using commercially available MEMSCAP based on PiezoMUMPS processes. In an anechoic chamber, the fabricated device has been excited in free-field sound, and the SSL at 1 kHz frequency, rocking frequency, bending frequency, and in-between rocking and bending frequencies has been found in full compliance with the given angle of incidence of sound. With the measured inter-aural sensitivity difference (mISD) and directionality, the developed BDM has been demonstrated as a practical SSL device, and the results have been found in a perfect match with the given angle of incidence of sound. Furthermore, to facilitate the SSL in noisy environment, the noise has been optimized in all scopes, like the geometry of the diaphragm, supportive torsional beam, and sensing. As a result, the A-weighted noise of this work has been found less than 23 dBA across the audio bands, and the equivalent-input noise (EIN) has been found to be 25.52 dB SPL at 1 kHz frequency which are the lowest ever reported by a similar device. With the developed SSL in broadband–in addition to the lowest noise–the developed device can be extended in some audio applications like an HA device.

## Introduction

The SSL is one of the fundamental requirements of some free–field and far–field acoustic applications, such as mobile robot, noise activated cameras in surveillance system, and HA^1^. The conventional approaches of SSL are reported using *time difference of arrival* (TDOA)^1,2^. Under this approach, the SSL can be modeled on two or more omnidirectional microphones by imitating wavelength of interest as the pre-defined inter-distance^2^; as a result, the whole sensory system becomes bulky in size, and most importantly, such devices suffer from high computation time, high noise, low signal–to–noise ratio (SNR)^1–3^.

**Figure 1:**
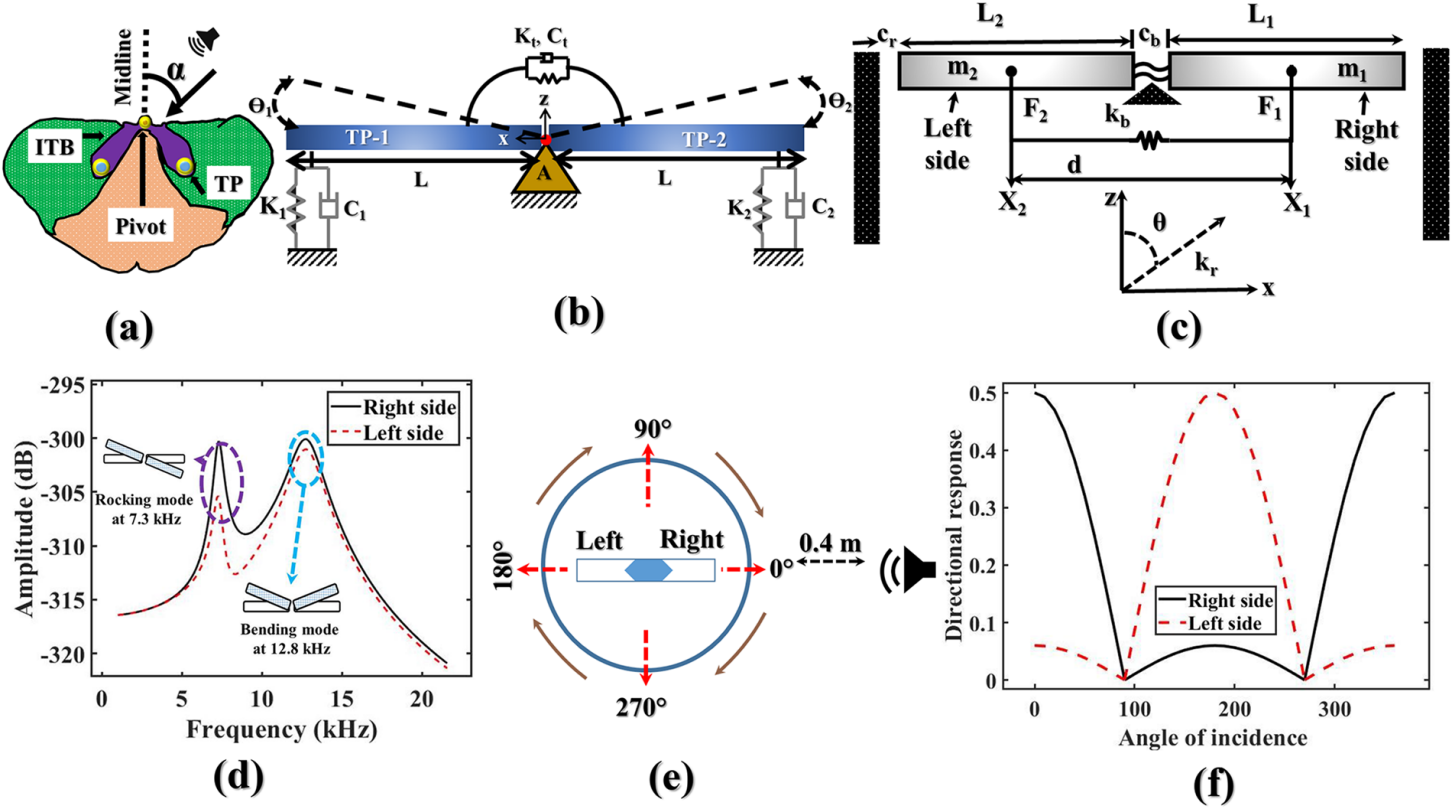
Modelling of bio–inspired MEMS directional microphone. (**a**) sketched of the ears of fly *Ormia ochracea*; where, *α* is the angle of incidence of sound, (**b**) mechanical model which was re-drawn from Miles *et al*.^4^; where, *θ*_1_, *θ*_2_, *K*_*t*_, *C*_*t*_, *K*_1_, *C*_1_, *K*_2_, *C*_2_, and L are the angle of rotation of tympanum (TP–1), angle of rotation of tympanum (TP–2), coupling’s spring, coupling’s damper, spring of TP–1, damper of TP–1, spring of TP–2, damper of TP–2, and identical length of both diaphragms, respectively, (**c**) modified mechanical model of this work; where, *m*_1_ = *m*_2_, *k*_*r*_, and *k*_*b*_ are the identical mass of both diaphragms, torsional stiffness, and bending stiffness, respectively, (**d**) analytical simulation in frequency domain, (**e**) sketched setup to model the directional response, and (**f**) analytical directivity response with varying angle of incidence from 0° to 360° at 7.3 kHz and 1 Pa sound pressure.

In contrast, the fly *Ormia ochracea*’s ears–inspired directional microphone is relatively better for SSL regarding its outperforming directionality, and low-internal noise at reduced size^2,4,5^. Figure 1(a) shows a sketched view of the fly’s hearing organ. The understanding of the ears of fly *Ormia ochracea* implies that: this parasitic fly has two tympana (TP) which are pivoted at the inter-tympanal bridge (ITB) with an inter-distance of 520 *μ*m^4–6^. Miles *et al*.^4^ reported the basis of mimicking the ears of fly *Ormia ochracea* using a spring-mass-damper system (see Fig. 1(b)). Figure 1(b) can be described as: when a sound pressure incidence on a tympanum, then both tympana show a phase difference relative to normal axis of the farthest tympanum^4^. As a result, they show a amplitude difference in form of directional cues, such as inter-aural time difference (ITD) & inter-aural intensity difference (IID)^4,6–8^. The cues are amplified from 1.5 *μ*s to 50 *μ*s and 1 dB to 12 dB, respectively for ITD, and IID near at the rocking mode which leads a SSL in range of ±30° with ±2° accuracy^6,8–10^. By inspiring these astonishing abilities of the fly *Ormia ochracea*, a number of SSL works have been reported, such as SSL at bending mode (1.69 kHz)^9^, rocking mode^6,8,10–12^, at 2 kHz^13^, low-frequency (below 3 kHz)^14^. Moreover, the majority of aforementioned works are fully replicated the ears of fly *Ormia ochracea*; as a result, they showed best performance at a single frequency like-wise the fly *Ormia ochracea*–best performance is at 5 kHz^4,5,15,16^. Therefore, the literature of the SSL using fly *Ormia ochracea*-inspired MEMS directional microphone leaves a lack of attaining SSL in broadband, or simultaneously at rocking and bending modes either.

In this paper, we report on a novel idea to achieve the SSL in broadband where the knowledge of sound pressure level (SPL) and distance of sound source with respect to the directional microphone are not required. Because the cosine dependency works for the SSL relative to the angular rotation of incoming sound; whereas, the magnitude difference followed by the phase difference between two pivoted diaphragms leads the SSL in frequency domain. Based on this understanding, we presented the SSL with varying azimuth angles from 0°–360° and frequency domain SSL in audio bands. To validate the credibility of this work, the SSL has been practically demonstrated both in frequency domain and relative to the angular rotation of the incoming sound. The work presented in this article is novel because it takes the advantages of mechanical coupling and cosine dependency to give wide-band SSL which is new and not complex as compared to the traditional fly *Ormia ochracea*-inspired SSL works. The outstanding contributions of this work as compared to the similar devices are as follows: (1) the SSL, in fact for the first time, in wide–band was modeled and experimentally presented by an Aluminum Nitride (AlN) & D33 mode based bio-inspired piezoelectric MEMS directional microphone, (2) unlike Kuntzman *et al*.^13^, a modified cosine dependency algorithm was adopted; which in turn, the issue of localizing the “off-axis” was solved in this work, and (3) for the first time, a MEMS directional microphone with less than 23 dBA broadband noise was developed and experimentally presented.

## Results

### Mathematical model

Figure 1(c) shows the modified mechanical model as compared to the basis model reported by Miles *et al*.^4^ where it can be noticed that two optimized identical diaphragms (right side and left side) were pivoted followed by the coupling mechanism of fly *Ormia ochracea*’s ear. The optimization was accepted in terms of model frequency within the audio band and low-noise across the audio bands from the prior work^17^ so that the developed device can mitigate the constraints of traditional works. The mass (m), length (L), acting force (F) and displacement (X) having subscript 1 are representing right side; whereas, the subscript 2 describes the left side. In addition, c_*r*_, c_*b*_, k_*r*_, k_*b*_, d, and *θ* are the damping coefficient at rocking mode, damping coefficient at bending mode, torsional stiffness, bending stiffness, distance between two force points, and angular rotation of the diaphragm, respectively. To give an insight of the coupling, the equations of motion of the mechanical model by assuming small angular bending can be given as^4,14,18^,

1 $$I\ddot{\theta }(t)+{c}_{r}\dot{\theta }(t)+{k}_{r}\theta (t)=d/2\times {f}_{1}(t)-d/2\times {f}_{2}(t)$$

2 $$[\begin{array}{ll}{m}_{1} & 0\\ 0 & {m}_{2}\end{array}]\,[\begin{array}{l}{\ddot{x}}_{1}(t)\\ {\ddot{x}}_{2}(t)\end{array}]\,+[\begin{array}{ll}{c}_{b} & 0\\ 0 & {c}_{b}\end{array}]\,[\begin{array}{l}{\dot{x}}_{1}(t)\\ {\dot{x}}_{2}(t)\end{array}]\,+[\begin{array}{ll}{k}_{b} & {k}_{b}\\ {k}_{b} & {k}_{b}\end{array}]\,[\begin{array}{l}{x}_{1}(t)\\ {x}_{2}(t)\end{array}]=[\begin{array}{l}{f}_{1}(t)\\ {f}_{2}(t)\end{array}]$$

where, I is the mass moment of inertia of whole diaphragm. Also, *f*_1_(*t*), and *f*_2_(*t*) are the acting forces in form of the product of acting pressure (P) and area of the each diaphragm (*A*_*d*_) as, *s* = *P* × *A*_*d*_. The transfer functions of forces are *F*_1_(*jω*) = *se*^*jwτ*/2^ and *F*_2_(*jω*) = *se*^−*jwτ*/2^; where, *τ* is the time delay followed by phase difference (*ϕ*) as, *τ* = *d*/*c* × *cos*(*ϕ*); where, *ϕ* = (*ω* × *d*)/*c* × *cos*(*α*) and *α* is the angle of incidence of incoming sound^4,11^. After applying Laplace transformation, Eqs. (1) and (2) are found to be^14^,

3 $$\theta (j\omega )=\frac{d/2\times {F}_{1}(j\omega )-d/2\times {F}_{2}(j\omega )}{I({\omega }_{r}^{2}-{\omega }^{2}+2j\omega {\omega }_{r}{\zeta }_{r})};\,{x}_{1(2)}=\frac{{F}_{1(2)}(j\omega )+\{({F}_{2(1)}(j\omega )-{F}_{1(2)}/{(\omega /{\omega }_{b})}^{2}\}}{2m({\omega }_{b}^{2}-{\omega }^{2}+2j\omega {\omega }_{b}{\zeta }_{b})}$$

where, *m*_1_ = *m*_2_ = m, ${\omega }_{r}=\sqrt{{k}_{r}/I}$, ${\omega }_{b}=\sqrt{{k}_{b}/m}$, *c*_*r*_ = 2*ω*_*r*_*Iζ*_*r*_, and *c*_*b*_ = 2*ω*_*b*_*Iζ*_*b*_ are the identical mass of both diaphragms, angular frequency of rocking mode, angular frequency of bending mode, damping coefficient at rocking mode, and damping coefficient at bending mode, respectively. Also, *ζ*_*r*_, and *ζ*_*b*_ are the damping ratios at rocking mode and bending mode, respectively which were calculated using measured quality factor followed by the foundry work on piezoelectric BDM^18^ (**see “Supplementary** **Table** **S1****”**). Then, the total displacement by each diaphragm can be given as,

4 $${X}_{1}={x}_{1}+d/2\times \theta (j\omega );\,{X}_{2}={x}_{2}-d/2\times \theta (j\omega )$$

The analytical simulated result is shown in Fig. 1(d). The analytical simulation was performed on Eq. (4) by assuming sound is coming from 0° of incidence (right side) and using parameters listed in **“**Supplementary Table S1**”**. It can be noticed that the magnitude of right side is constantly higher than the left side due to the phase difference followed by mechanical coupling^4^. With this magnitude difference, the location of sound source can be easily detected. The inset of Fig. 1(d) shows the rocking mode, and bending mode, respectively due to the out-of phase, and in-phase positions of both diaphragms^4^. In the analytical model, the rocking mode frequency (f_*r*_) was found at 7.3 kHz governed by the torsional stiffness (*k*_*r*_) and mass-moment of initial of whole diaphragm (I) as, ${f}_{r}=1/2\pi \times \sqrt{{k}_{r}/I}$^19^. Whereas, the bending mode frequency (f_*b*_) was appeared at 12.8 kHz followed by the bending stiffness (*k*_*b*_) and mass (m) as, ${f}_{b}=1/2\pi \times \sqrt{{k}_{b}/m}$^19^. The parameters value behind the calculation of modal frequencies can be found in **“Supplementary** **Table** **S1****”**.

One step further, the directional response relative to the angular rotation of incoming sound was modeled on Fig. 1(e). The arrow in Fig. 1(e) shows the rotation of the developed BDM while the sound source is fixed at 0.4 m apart from the BDM. At 0°–360° rotation the directional response of the coupled diaphragm can be given as^20^,

5 $${V}_{r}=\mathop{\underbrace{\frac{1}{2}P(\alpha ,\omega )|cos(\alpha )|}}\limits_{{\rm{Higher}}\,{\rm{at}}\,{90}^{\circ } < \alpha { > 270}^{\circ }}+\mathop{\underbrace{\frac{1}{2}P(\alpha ,\omega ){\beta }_{r}(\omega ,\phi )|cos(\alpha )|}}\limits_{{\rm{Lower}}\,{\rm{at}}\,{90}^{\circ } > \alpha { < 270}^{\circ }}$$

where, *V*_*r*_, 1/2, P(*α*, *ω*), and *β*_*r*_(*ω*, *ϕ*) are the right side’s directional response, half area, applied sound as a function of angle of incidence (*α*) and angular frequency in radian/s, and delay of right side due to phase difference (*ϕ*) for the farthest diaphragm position at 90° > *α* < 270°, respectively. In the case of *V*_*r*_, the first part of Eq. (5) is for the rotation within 90° < *α* > 70°; where, the right side is the closest diaphragm relative to the sound source; as a result, it gives higher response. Also, the second part of Eq. (5) is lagging by delay factor (*β*_*r*_) at 90° > *α* < 270° due to farthest position relative to the sound source. On the other hand, when the right side goes at the farthest position, then the left side becomes closest relative to the sound source. The directional response of left side (*V*_*l*_) can be given as^20^,

6 $${V}_{l}=\mathop{\underbrace{\frac{1}{2}P(\alpha ,\omega ){\beta }_{l}(\omega ,\phi )|cos(\alpha )|}}\limits_{{\rm{Lower}}\,{\rm{at}}\,{90}^{\circ } < \alpha { > 270}^{\circ }}+\mathop{\underbrace{\frac{1}{2}P(\alpha ,\omega )|cos(\alpha )|}}\limits_{{\rm{Higer}}\,{\rm{at}}\,{90}^{\circ } > \alpha { < 270}^{\circ }}$$

where, *V*_*l*_, and *β*_*l*_(*α*, *ϕ*) are the left side’s directional response, and delay of left side due to phase difference (*ϕ*) for the farthest diaphragm position at 90° < *α* > 270°, respectively. Figure 1(f) shows the directional response of right and left diaphragms at a randomly chosen frequency (7.3 kHz). It can be noticed in Fig. 1(f) that the right side gives higher response at 90° < *α* > 270° as we expected by Eq. (5). On the other hand, when the left side becomes prominent at 90° > *α* < 270° the left side gives higher directional response than the right side expected by Eq. (6).

Furthermore, to present the SSL experimentally, the designed device was fabricated by a commercially available Multi-users MEMS processes (MUMPs), i.e., PiezoMUMPs through MEMSCAP Inc.^21^. **“Supplementary information section 2”** gives an insight of the fabrication along with the formation of the D33 mode and **“Supplementary** **Table** **S1****”** shows the device parameters. In short, to convert the mechanical vibration into electrical signal, a piezoelectric sensing was chosen due to its flexibility, i.e., no bias-voltage needed, easy to handle and most importantly low-noise as compared to the capacitive sensing^19^. Moreover, among the available piezoelectric materials, the Aluminum Nitride (AlN) gives low acoustic loss, dielectric loss tangent and compatibility with CMOS circuits which makes it better candidate to control the electronic noise^19^. Finally, the AlN has been operated in 3–3 stress–strain directions to enhance the sensing signal^22^. The combination of AlN and D33 mode is less explored and has two fundamental merits, such as low dielectric loss (0.002)^23^ which minimizes the thermal-electrical noise^24^, and higher electrode spacing (user–defined) as compared to D31 mode which enhances sensitivity^19,22^. As a result, this combination can give higher SNR which minimizes the equivalent–input noise which will be discussed further in **“Noise optimization”**.

### Experimental measurement of SSL

**Figure 2:**
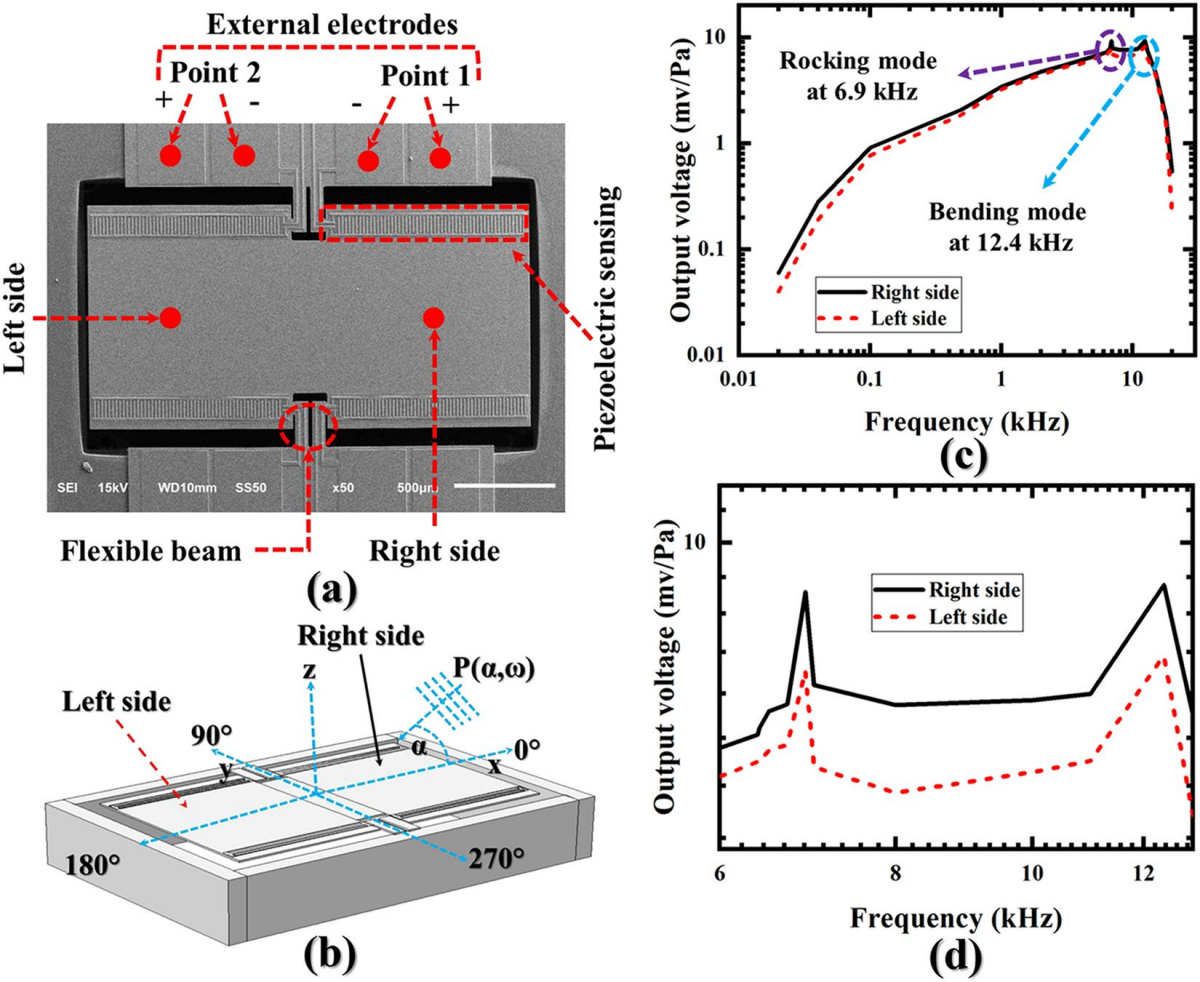
Frequency response of both diaphragms of the developed piezoelectric BDM. (**a**) SEM of the fabricated device, (**b**) schematic of the experimental setup; where, P(*α*, *ω*) is the sound wave incidences on the right side (x–z plane) from 0° of incidence, (**c**) frequency response of both diaphragms at 1 Pa/94 dB SPL sound pressure and 0° of incidence of sound with varying audio frequencies from 20 Hz to 20 kHz, and (**d**) zoomed view of the frequency response of both diaphragms at 0° of incidence of sound having 1 Pa/94 dB SPL pressure with varying frequencies from 6 kHz to 13 kHz.

Figure 2(a) shows the scanning electrode micrograph (SEM) of the fabricated device; where, the external electrodes belong to right side are denoted as **“Point 1”**; whereas, left side is denoted as **“Point 2”**. In an anechoic chamber, the fabricated device was excited in free-field space at 94 dB sound pressure level with varying audio frequencies and azimuth angles depending on the experiments. The details on the experimental setup will be discussed in the **“Experimental measurement”** section. A schematic of the setup is shown in Fig. 2(b) where it can be noticed that the sound incidences in the x-z plane from the right side of the BDM.

Figure 2(c) shows the frequency response of the developed BDM at 94 dB SPL sound pressure with varying audio frequencies. To carry out the measurement, the sound was applied from the 0° of incidence as shown in Fig. 2(b). The measured rocking frequency was found at 6.9 kHz which is 5.5% deviated from the analytically simulated rocking frequency, and bending frequency was appeared at 12.4 kHz which is 3.4% deviated from the analytically simulated bending frequency (see Fig. 1(d)). Moreover, the purpose of the frequency response is to detect the sound source in frequency domain which largely depends on the measured inter-aural sensitivity difference (mISD). Therefore, the basic characteristics of the frequency response were not discussed in the main text, however, they can be found in our prior work^17,19^ and also in **“Supplementary Table** **S2****”**. To present the mISD, Fig. 2(c) was zoomed and re-plotted from 6 kHz to 13 kHz frequency which shown in Fig. 2(d). It can be noticed that the right side is constantly giving higher response than the left side as we mechanically demonstrated by Eq. (4). It should be noted that the Fig. 1(d) deals only with the mechanical vibrations of the coupled diaphragms; whereas, Fig. 2(c,d) show the electrical signal, thus, the signal levels are not comparable to each other. With this sufficient sensitivity difference, the location of the sound source can be easily estimated using a simple subtraction logic as^13^, *mISD* = *V*_*r*_ − *V*_*l*_; where, *V*_*r*_ is the right side’s response and *V*_*l*_ is the left side’s response. The positive polarity of mISD identifies that the right side response is higher due to nearest position to the sound source. On the other hand, the negative polarity identifies that the sound is coming from left side. In **“Practical demonstration of SSL”** section, an algorithm based on the mISD will be adopted to display the SSL in frequency domain.

**Figure 3:**
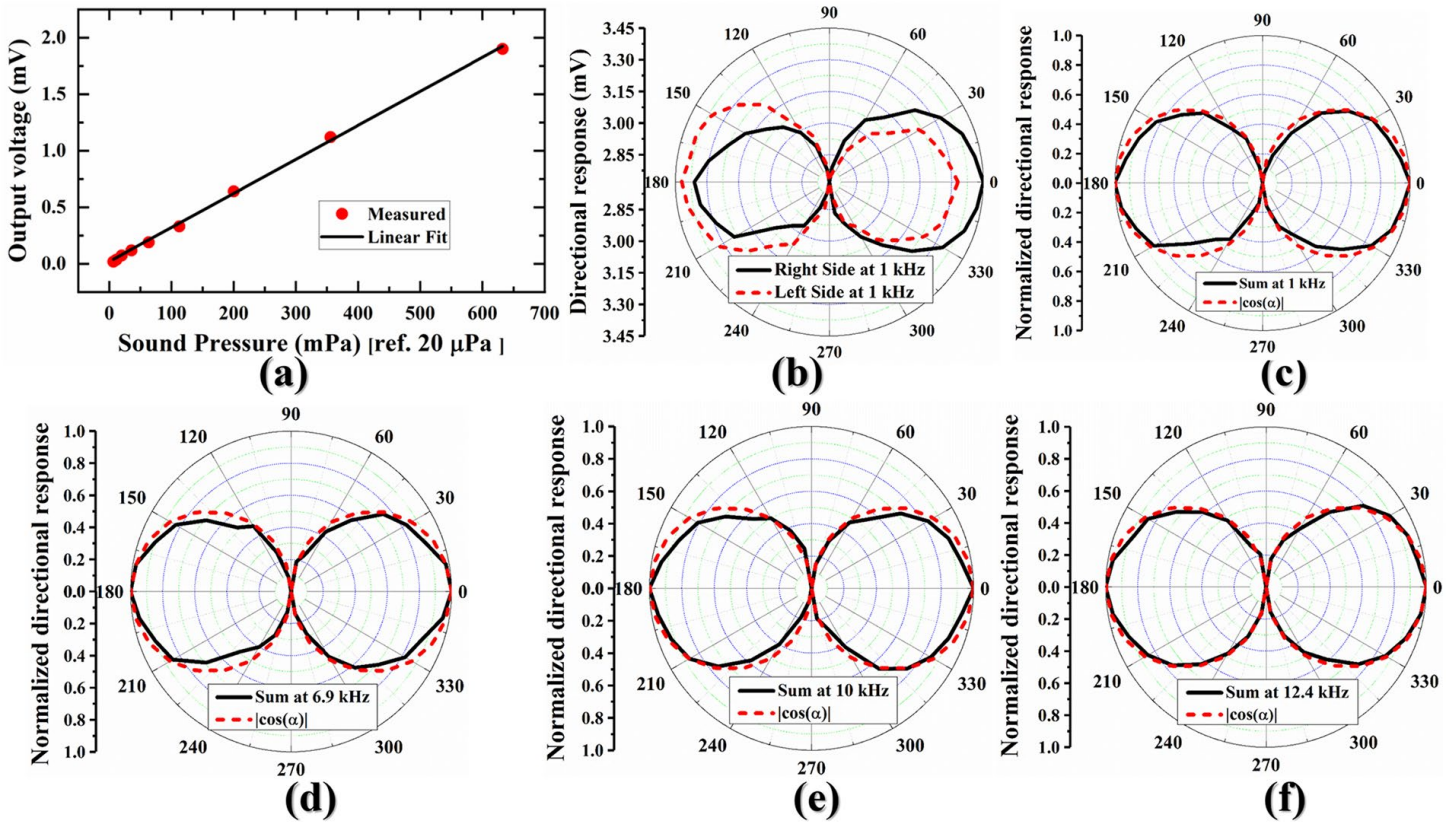
Pressure sensing and directionality measurements. (**a**) sensing response with varying sound pressures from 50 dB SPL to 90 dB SPL at 1 kHz frequency, (**b**) directional response of both diaphragms at 1 kHz frequency and 1 Pa sound pressure with varying azimuth angles from 0° to 360°, (**c**) summed directional response of both diaphragms at 1 kHz and 1 Pa sound pressure along with the directional response of an ideal pressure–gradient sensor, (**d**) summed directional response of both diaphragms at rocking mode (6.9 kHz) and 1 Pa sound pressure with a comparison to an ideal pressure–gradient sensor, (**e**) summed directional response of both diaphragms at 10 kHz and 1 Pa sound pressure with a comparison to an ideal pressure–gradient sensor, and (**f**) summed directional response of both diaphragms at bending mode (12.4 kHz) and 1 Pa sound pressure with a comparison to an ideal pressure–gradient sensor.

Figure 3(a) shows the sensing response of the developed BDM at 1 kHz frequency with varying sound pressures from 50 dB SPL to 90 dB SPL. To do the measurement, at first, the sound pressure of the anechoic chamber was measured using a digital sound level meter (GM1351, Digital sound level meter), and it was found to be ~37–43 dB SPL depending on the inherent sound of the measuring devices. Therefore, the applied sound pressure was varied from 50–90 dB SPL in a sense to avoid the inherent sound of the measuring devices. Under 50 dB SPL to 90 dB SPL variations which are equivalent to 6.32 mPa to 632.46 mPa [pressure ref. 20 *μ* Pa]^25^, the output voltages were found from 0.0018 mV to 1.9 mV, which implies a linear dependency on the given sound pressure. With this variations of the sound pressure, the measured sensitivity was found to be 3.45 mV/Pa at 94 dB SPL sound pressure.

Figure 3(b) shows the measured directional response of the developed BDM at 1 kHz frequency and 94 dB SPL sound pressure with varying azimuth angles from 0°–360° with an interval of 10°. It can be noticed that the right side’s response (black solid line) is constantly higher at 90° > *α* > 270°; whereas, the same side’s response is lagging by delay factor (*β*_*r*_) at 90° < *α* < 270° as we mechanically demonstrated by Eq. (5). On the other hand, when the BDM is further rotating and left side becomes prominent; as a result, the left side gives higher response (red dashed line) at 90° < *α* < 270° as we mechanically demonstrated by Eq. (6). To understand how these responses play the role for SSL in azimuth angles, both response were summed and compared with an ideal pressure gradient microphone. It is noted that the linear summation was used followed by the foundry work on piezoelectric BDM^18^. The summation of right and left side’s response can be found by using Eqs. (5) and (6) as^18^,

7 $$V=P(\alpha ,\omega )|\,\cos (\alpha )|\left[1+\frac{1}{2}\{{\beta }_{r}(\alpha ,\phi )+{\beta }_{l}(\omega ,\phi )\}\right]=P(\alpha ,\omega )\beta |\,\cos (\alpha )|$$

where, $\beta =1+\frac{1}{2}\{{\beta }_{r}(\alpha ,\phi )+{\beta }_{l}(\omega ,\phi )\}$ is the total delay factor for right and left sides of the developed BDM depending on their farthest position relative to the sound source. Also, V is the summed response of the right side’s response (*V*_*r*_) and left side’s response (*V*_*l*_). Analytically, Eq. (7) is the general expression of an ideal pressure–gradient sensor as we expected by keeping the directional microphone’s backside open. Figure 3(c) shows the normalized summed response of 1 kHz frequency and 1 Pa sound pressure (taken from Fig. 3(b)) along with the the simulation of an ideal pressure-gradient sensor. The simulation parameters can be found in **“Supplementary** **Table** **S1****”**. Furthermore, the directivity response was extended towards rocking mode, bending mode, and in-between rocking and bending modes. Figure 3(d–f) show the directivity responses at rocking mode frequency (6.9 kHz), in-between rocking and bending modes (10 kHz), and bending mode frequency (12.4 kHz), respectively. It can be noticed in Fig. 3(c–f) that all directivity measurements are in a good match with the simulated response of an ideal pressure-gradient sensor.

### Formation of SSL

Equation (7) can be treated with maximum sensing signal at 0°/180° (see Fig. 3(b)) and minimum value at 90°/270° (see Fig. 3(b)) to detect each incidence angle of incoming sound. By assuming, the signal at 90°/270° as *V*_*off*_, the expression for the detection of angle of incidence of incoming sound can be found to be^8,13^,

8 $${\alpha }_{m}={\cos }^{-1}\left(\frac{{V}_{m}-{V}_{off}}{V-{V}_{off}}\right)$$

where, *α*_*m*_, and *V*_*m*_ are the measured angle and measured sensing response at a given angle of incidence, respectively. A new form of directionality measurement was taken into account where the angular rotation was performed in 30° of interval and the measured response was treated using Eq. (8). Table 1 shows the results at 1 kHz frequency and 94 dB SPL. To do that, at first, the directional response of right side was measured which are shown in second column of Table 1. Then, the left side’s response was measured which are listed in third column of Table 1. After having two sides responses, both responses were summed followed by Eq. (7) and then treated with Eq. (8). It can be noticed that all the measured angles were found in a good match with the given angle of incidences.

**Table 1:** Sound source localization at 1 kHz and 94 dB SPL using developed bio-inspired piezoelectric directional microphone.

Given angle *α*°	Right diaphragm V_*r*_ (mV)	Left diaphragm V_*l*_ (mV)	Summed response V = V_*r*_ + V_*l*_ (mV)	Measured angle *α*_*m*_°	Differences |*α*° − *α*_*m*_°|
0	3.45	3.33	6.78	0	0
30	3.33	3.22	6.55	33.9	3.9
60	3.06	2.90	5.96	67.05	7.05
90 (V_*off*_)	2.72	2.72	5.44	90	0
120	2.99	3.03	6.02	115.46	4.54
150	3.18	3.36	6.54	146.09	3.91
180	3.33	3.45	6.78	180	0
210	3.24	3.33	6.57	211.79	1.79
240	2.96	3.06	6.02	243.9	3.9
270 (V_*off*_)	2.72	2.72	5.44	270	0
300	3.02	2.96	5.98	293.58	6.42
330	3.34	3.24	6.58	328.21	1.79
360	3.45	3.33	6.78	360	0

**Figure 4:**
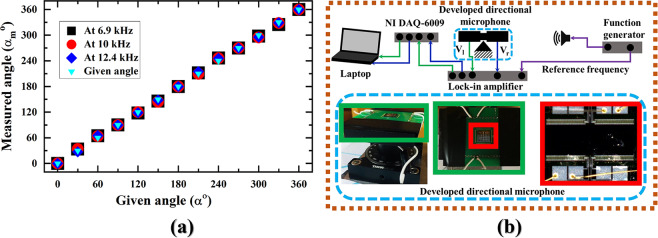
Formation and display of SSL. (**a**) SSL in azimuth angles varying from 0°–360° at 6.9 kHz, 10 kHz, and 12.4 kHz frequencies and 1 Pa sound pressure, and (**b**) a complete setup in anechoic chamber to display the SSL in both frequency domain and azimuth angle.

One step further, Fig. 4(a) shows an extension of SSL in azimuth angles at rocking frequency (6.9 kHz), bending frequency (12.4 kHz), and an extension to understand the effect of rocking frequency & bending frequency (10 kHz). All measured angles are in a good match with the given angle of incidence which verifies the cosine dependency of the developed bio-inspired directional microphone.

### Practical demonstration of SSL

For the proof-of-concept, the SSL in frequency domain and SSL in angular rotation of sound were merged together in Fig. 4(b) to perform the practical demonstration. The Fig. 4(b) itself has three sections, such as directional microphone, processing electronic circuitry, and logic interface with the personal computer. The extended view of Fig. 4(b) can be found in **“Fig.** **S2** **in supplementary information section 3”** where it shows how we installed the setup in anechoic chamber to perform the demonstration. Under a given sound, the each diaphragm of the directional microphone generates electrical signal (voltage) by piezoelectricity effect, namely *V*_*r*_ from the right side, and *V*_*l*_ from the left side. In next phase, the output response of each diaphragm was processed through a lock-in amplifier (SR830, Stanford Research Systems), and then the signal was interfaced to LabVIEW 2015 version software using a data acquisition device (USB-DAQ 6009, National Instruments). In LabVIEW, the signal from each diaphragm was handled by two logic, such as detection of the source using the mISD, and localization of incoming sound using cosine dependency. To detect sound, three conditions were made in LabVIEW which are: (1) V_*r*_-V_*l*_ > 0: when the response of the right side is higher due to the closest position with respect to the sound source, i.e., the 0° incidence of sound (see Fig. 2(b)), (2) V_*r*_ –V_*l*_ = 0: when sound arrives at the coupling area (i.e., from 90°/270° of incidence), and (3) V_*r*_ –V_*l*_ < 0: the response of the left side is higher due to the closest position with respect to the sound source, i.e., the 180° incidence of sound. A demonstration was performed until this point, and associate video can be found in the **“SV_****2.mp4****” in** **supplementary** **files**. In the movie file, it can be noticed that each condition to detect the sound source was successfully performed. Then, after detecting the closest diaphragm with respect to the sound source, the signal of closest diaphragm was passed through the second logic to localize the incidence angle of the sound. The logic was made using the cosine factors for each given angle. A demonstration was performed, and **“SV**_**3**.**mp4****” in** **supplementary** **files** shows the associate results. In the video file, it can be noticed that we successfully localized the incidence angle of the given sound.

### Noise optimization

**Figure 5:**
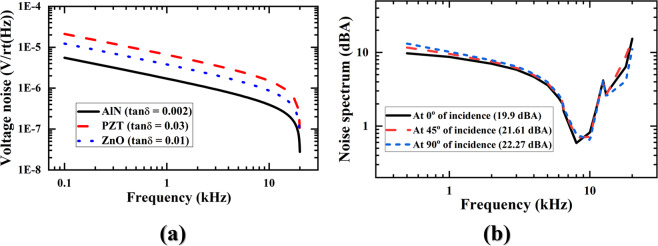
Broadband noise analyses of the developed bio-inspired piezoelectric MEMS directional microphone. (**a**) Voltage noise varying with dielectric loss (tan *δ*) of several piezoelectric materials, and (**b**) A–weighted noise under full audio frequency.

To employ the developed SSL in noisy environment, we take a look at the noise optimization so that the noise can be controlled as much as possible. It is a well-known fact that the main noise contributors of MEMS microphones are the sensor itself^24,26,27^, and the processing circuitry^23,25^. The sensor noise largely depends on the geometry of the sensor^17,26^ and sensing system^17–19^; whereas, the noise due to the processing circuitry can be about 3 dB^26^ using a fine tuning measuring devices. In our previous work^17^, we reported the effect of the device dimensions including diaphragm and supportive torsional beam on signal degradation, and then, the optimized torsional beam as well as dimensions of the diaphragm was proposed to attain low–noise^17^. It was found from the previous study^17^ that a 225 *μ*m torsional beam with 1770 *μ*m × 1100 *μ*m (total length (*L*_1_ + *L*_2_), width) diaphragm can provide 25.67 dB SPL equivalent–input noise at 1 kHz frequency only when the sensing is designed with AlN and D33 mode. Because, this noise depends on the SNR of the device and the SNR is the outcome of the voltage noise and sensitivity. The use of AlN minimizes the main noise contributor, i.e., thermal-electrical noise (voltage noise). From “**Fig.** **S3** **in the supplementary information section 4**.**1**”, the voltage noise can be derived as^24^, $\sqrt{4{K}_{b}T{R}_{m}}$; where, *k*_*b*_, T, and *R*_*m*_ are the Boltzmann constant, room temperature, and resistance respectively. Meanwhile, the resistance (*R*_*m*_) can be defined as^24^, ${R}_{m}=\frac{\tan \,\delta }{\omega \times {C}_{eb}}$; where, *ω*, tan*δ*, and *C*_*eb*_ are the frequency in radian/s, dielectric loss tangent of the piezoelectric material, and blocking capacitance, respectively. The formation of (*R*_*m*_) implies that the dielectric loss tangent can catalyze the voltage noise. Figure 5(a) shows the voltage noise of the AlN, lead zirconate titanate (PZT), zinc oxide (ZnO), respectively for 0.002^23^, 0.03^23^, 0.01^23^ dielectric loss tangent. It can be noticed in Fig. 5(a) the AlN is better piezoelectric material to minimize noise. On the other hand, the piezoelectric coupling of D33 mode is higher than D31 mode^24^, and it is a well-known fact. With the higher piezoelectric coupling–in addition to the higher electrode spacing–the D33 mode enhances the sensing signal. As a result, the SNR improves, and the improved SNR minimizes the equivalent–input noise (EIN) by EIN = 94-SNR (dB SPL). In this work, the measured EIN was found to be 25.52 dB SPL made of 68.47 SNR, which is 0.62% deviated from the analytical prediction, i.e., 25.68 dB SPL (see **“Supplementary** **Table** **S2****”**).

Figure 5(b) shows the A-weighted noise spectrum of this work under full audio frequency at 0°, 45°, and 90° of incidences of sound with respect to the right diaphragm. **“Equations** (S2)–(**S3)** **in the supplementary information section 4**.**2”** were used to derive the A-weighted noise. The derived noises were found to be 19.9 dBA, 21.61 dBA, and 22.27 dBA respectively for 0°, 45°, and 90° angle of incidence of sound.

## Discussion

Hearing Aids (HA) is the largest application area of the directional microphone. However, the works intended to be using in HA were limited to single frequency of operation/narrow–band which leave a limited choice at the user’s end. As a result, despite of having better directionality and noise performance, the bio–inspired directional microphones are not getting that much attention to be implemented. Moreover, some of them were not even fit to localize certain angle of incidences. For instance, Kuntzman *et al*.^13^ reported the basis of the SSL using the fly *Ormia ochracea*’s ears–inspired piezoelectric MEMS directional microphone. However, the work reported by Kuntzman *et al*.^13^ was limited to 2 kHz frequency, and most importantly, the “off-axis” response was not included in the SSL model, which in turn, showed a lack to localize 90° of incidence of sound.

To this particular purpose, we present a novel idea where, the developed BDM takes advantages of coupling and gives a magnitude difference to identify the sound source. In the follow through, the developed device was analytically simulated to see the mechanical behaviour under sound. Moreover, in the experimental measurements, the developed device showed a similar behaviour as compared to an ideal pressure–gradient sensor. It should be noted that a modified cosine algorithm was adopted to localize all the angle of incidence of incoming sound; as a result, the developed device showed outperforming characteristics as compared to the foundry work on SSL using similar device by Kuntzman *et al*.^13^. Moreover, **“supplementary** **Table** **S3****”** shows a point–to-point comparison between this work and the foundry work by Kuntzman *et al*.^13^. Besides the SSL, the optimization of the device leaded a tremendous noise control as compared to the *state–of–the–art* of similar device. Table 2 shows a comparison between this work and some notable works inspired by the ears of fly *Ormia ochracea*; where, it can be clearly seen that the developed device not only satisfies the wide–band SSL, but also gives lowest noise ever reported by the similar device.

**Table 2:** Comparison of findings between this work and some notable works inspired by the ears of fly *Ormia ochracea* with similar applications.

Works	Sensing	SSL formation	Working frequency (kHz)	Noise floor (dBA)
Miles *et al*.^2^	Optical	Cosine	0.8	35.6
Liu *et al*.^6^	Optical	Mechanical phase & time differences	8	—
Miles *et al*.^27^	Capacitive (Comb finger)	Cosine	<3	43.1
Kuntzman *et al*.^9^	Piezoelectric (PZT&D31 mode)	Cosine	2	42
Wilmott *et al*.^18^	Capacitive (Comb finger)	Cosine	1.69	—
Zhang *et al*.^14^	Piezoelectric & Capacitive	Cosine	<3	—
**This work**	**Piezoelectric** (**AlN&D33 mode**)	**Modified cosine**	**1–13**	**<23**

Besides the A-weighted noise, the equivalent–input noise (EIN) was found to be 25.52 dB SPL at 1 kHz frequency which is same as the human hearing threshold^18^. According to the foundry work on fly *Ormia ochracea*’s ears–inspired microphone^27^, the noises, such as EIN and A-weighted largely depends on the coupling and diaphragm dimensions. With this hint, in our previous work^17^, we looked at the optimizations, and the best dimensions were adopted for this work. **“Supplementary** **Table** **S2****”** shows the comparison between the measured and predicted values of some basic acoustic functionalities like sensitivity, and noise. Moreover, to set the working frequency, we demonstrate the SSL in four different bands upto 13 kHz, and the results were found in a good match with the given angle of incidence of sound. However, the maximum difference was found to be 7.05° (see Table 1) at 1 kHz frequency. Whereas, the differences become lower with increasing frequency due to higher mISD, i.e., 6.42° at rocking mode, 6.42° in-between rocking and bending modes, and 5.8° at bending mode. However, all these differences were found at 60°, 120° and 240° of incidences. Thus, the reasons of the resolution change could be due to the low mISD at mismatch in rotation with respect to the sound, and fabrication tolerance. Combining all the outlines of this work, the developed work can be extended as a sound source localization device or HA. Therefore, the future work encloses with the packaging and clinical trial to be extended as a practical applications like HA.

## Methods

### Experimental measurement

**Figure 6:**
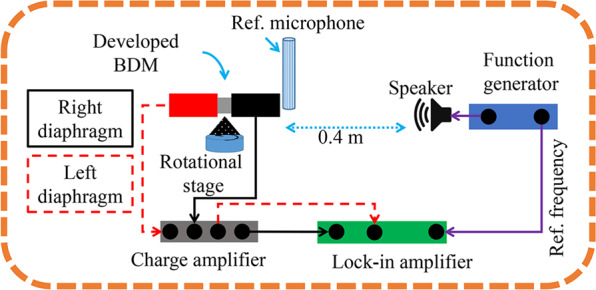
Experimental setup in an anechoic chamber to carry out all the experimental measurements of this work.

In this work, all the experimental measurements were carried out in an anechoic chamber using the experimental setup shown in Fig. 6. To make the setup, at first, the external electrodes of the directional microphone (see Fig. 2(a)) was connected to a custom printed circuit board (PCB) using a micro-wire bonder (4522, Kulicke & Soffa). The custom PCB along with the fabricated directional microphone was mounted on rotation state (Thorlabs Inc.) which has enough height to avoid the reflection from the surface (see **“Supplementary** **Fig.** **S2(a)****”**). On the other hand, the developed directional microphone was mounted horizontally on a rotational stage in a sense to avoid the back reflection of the custom PCB. The horizontal placement ensured the rotation of the directional microphone in normal axis with respect to the sound source. Then, the output terminal of the custom PCB were connected to a charge amplifier (SR570, Stanford Research Systems) using co-axial cable to avoid the cross-talk^22^. The sensitivity gain setting of the charge amplifier was 1 × 10 *μ*A/V with a 6 dB slope low-pass filter. Then, the output of the charge amplifier was connected to a lock-in amplifier (SR830, Stanford Research Systems) which was tuned to a 5 × 100 mV nA gain and a 3 × 100 ms time constant. On the other hand, the sound was generated using a function generator (SR345, Stanford Research Systems), and the sound pressure was calibrated using a reference microphone–B&K 4138-a pressure field microphone which was placed vertically near the developed directional microphone. The further calibration during the measurement of Fig. 3(a) was adopted using a digital sound level meter (GM1351, Digital sound level meter) to be sure with the sound pressure level (SPL). Notably, in all the measurements, a 1 Pa/94 dB SPL sound pressure was used.

## Supplementary information

Supplementary information.pdf.

SV_2.mp4.

SV_3.mp4.

## Data Availability

The datasets generated during and/or analysed during the current study are not publicly available due to confidential purpose but are available from the corresponding author on reasonable request.
