# Supplementary material for: Sound source localization by Ormia ochracea inspired low–noise piezoelectric MEMS directional microphone
Source: Sci Rep. 2020 Jun 12;10:9545. doi: 10.1038/s41598-020-66489-6 (PMC7293328; doi:10.1038/s41598-020-66489-6)
Supplement: Supplementary file 1 — Supplementary information.pdf. [file 41598_2020_66489_MOESM1_ESM.pdf]

Supplementary Information

Sound source localization by *Ormia ochracea* inspired low-noise piezoelectric MEMS directional microphone

Ashiqur Rahaman[1] and Byungki Kim[1,*]

[1]School of mechatronics Engineering, Korea University of Technology and Education, Cheonan 31253, Republic of Korea

*Corresponding author: byungki.kim@koreatech.ac.kr

CONTENTS

1. Supplementary Tables: 2

1.1. Table S1 2

1.2. Table S2 3

1.3. Table S3 3

2. Fabrication and device parameters: 3

3. Experimental setup: 5

4. Noise calculation: 6

4.1. Noise model: 6

4.2. A-weighted noise: 6

5. References 7

1. Supplementary Tables:

1.1. Table S1

Table S1. Designed and simulated parameters of this work.

**Symbol**	**Description**	**Value**	**Unit**
P	Applied sound pressure	1	Pa
$w_{t1=}$ w_{t2}	Width of the torsional beam	65	µm
l_t	Length of the torsional beam	225	µm
t	Thickness	10.7	µm
$J_{1(2)} = \left(\frac{1}{3} - 0.2\frac{t}{w_{t1(2)}}\right) w_{t1(2)} t^3$ **[SR_1]**	Torsional constant	23921	μm^4
ν	Poisson's ratio	0.28	N/A
E	Young's modulus	160	G Pa
G	Shear modulus	62.5×10^3	kg/µms^2
$k_{r1(2)} = \frac{J_{1(2)}G}{l_t}$ **[SR_1]**	Torsional stiffness	6644723	kg.µm^2/s^2
m	Mass	4.8×10^{-8}	Kg
$L=L_1=L_2$	Length of each diaphragm	885	µm
I	Mass moment of inertia	0.0125316	Kg.µm^2
[a] $f_r = \frac{1}{2\pi} \times \sqrt{\frac{k_{r1}}{I}} + \frac{1}{2\pi} \times \sqrt{\frac{k_{r2}}{I}}$ **[SR_1]**	Rocking frequency	7328	Hz
W	Width of the diaphragm	1100	µm
$k_b = \frac{E \times W \times t^3}{L^3}$ **[SR_2]**	Bending stiffness	311.05	kg.µm^2/s^2
$f_b = \frac{1}{2\pi} \times \sqrt{\frac{k_b}{m}}$ **[SR_2]**	Bending frequency	12811	Hz
d	Distance between two forces	970	µm
A_d	Area of each diaphragm	9.7×10^{-7}	m^2
f_r	Measured rocking frequency	6.9	kHz
Δf_r	-3 dB bandwidth of rocking mode	600	Hz
$Q_r=f_r/ \Delta f_r$ **[SR_3]**	Q-factor at measured rocking mode	11.5	N/A
$\xi_r=1/2\ Q_r$ **[SR_3]**	Damping ratio at rocking mode	0.04347	N/A
f_b	Measured bending frequency	12.4	kHz
Δf_b	-3 dB bandwidth of bending mode	2100	Hz
$Q_b=f_b/ \Delta f_b$ **[SR_3]**	Q-factor at measured bending mode	5.9	N/A
$\xi_b=1/2\ Q_b$ **[SR_3]**	Damping ratio at bending mode	0.08468	N/A
e_l	Length of main electrodes	760	µm
e_d	Main electrode gap	90	µm
e_w	Width of main electrodes	10	µm
i_l	D33 electrode's length	85	µm
i_w	D33 electrode's width	6.2	µm
i_s	D33 electrode's spacing	11.76	µm

[a] For two torsional beams

1.2. Table S2

Table S2: Comparison between measured results of this work and predicted results from our previous work **[SR_1]**.

Parameters	Analytical Model [SR_1]	Experimental Results
Sensitivity (mV/Pa)	4.58	3.45
EIN (dB SPL)	25.6	25.52
SNR	68.4	68.47

1.3. Table S3

Table S3. Comparison between this work and **Kuntzman et al. 2014 [SR_4].**

Parameters	Kuntzman et al. 2014 [SR_4]	This work
Diaphragm	Mechanical motion limited by the spring's elasticity	Able to achieve higher mechanical motion due to the free end of the diaphragm
Sensing	PZT and D31:<br>PZT: Higher dielectric loss tangent.<br>D31: Sensing signal limited by PZT thickness	AlN and D33:<br>AlN: Lower dielectric loss tangent as compared to PZT.<br>D33: User defined electrode spacing enhances the sensitivity.
Sensor noise	Higher sensor noise due to high dielectric loss tangent as shown in Figure 5 (a) in the main article.	Lower sensor noise due to low dielectric loss tangent as shown in Figure 5 (a) in the main article.
SSL	Outfit to localize 90° incidence of sound.	Measured and demonstrated 90° incidence of sound.
Presented SSL	2 kHz	1kHz, rocking mode (6.9 kHz), in-between rocking and bending modes (10 kHz), and bending mode (12.4 kHz)
A-weighted Noise	42 dBA **[SR_3]**	<23 dBA

2. Fabrication and device parameters:

The designed bio-inspired piezoelectric MEMS directional microphone was fabricated using a commercially available Multi-users MEMS processes (MUMPs) through PiezoMUMPs-an extension of the MEMSCAP Inc **[SR_5]**. Fig. S1(a) shows the cross-sectional view (not to scale); where AA´ is the cross-section line shown in Fig. S1(a) and Fig. S1(b). The fabrication starts with an n-type double side polished Silicon-On-Insulator (SOI) wafer which has 150 mm diameter with (100) lattice orientation **[SR_5]**. The wafer is composed of a 400 μm-thick handling substrate, a 1 μm-thick Oxide, and a 10 μm-thick Silicon. Then, the top surface of the Silicon layer was doped using phosphosilicate glass (PSG) layer and annealed at 1050 °C for 1 hour in Argon. Next, the PSG layer was washed out using chemical wet processing. After that, a 0.2 μm thermal oxide was grown on top of the Silicon layer which defined as the Pad

Oxide in Fig. S1(a). Then, a 0.5 μm-thick aluminum nitride (AlN) was patterned, wet etched followed by a solvent resist strip. Next, on top of the AlN layer, a 0.02 μm-thick chrome and a 1 μm-thick aluminum were patterned using the liftoff process **[SR_5]**. Then, the combination of chrome and aluminum was patterned as D33 electrodes which is defined as electrode which shown in Fig. S1(a). Finally, with the top surface protection, the wafers were rotated in reversed and the bottom side oxide layer was etched using Reactive Ion Etching (RIE). The further details on the fabrication can be found in the manual of PiezoMUMPs **[SR_5]**.

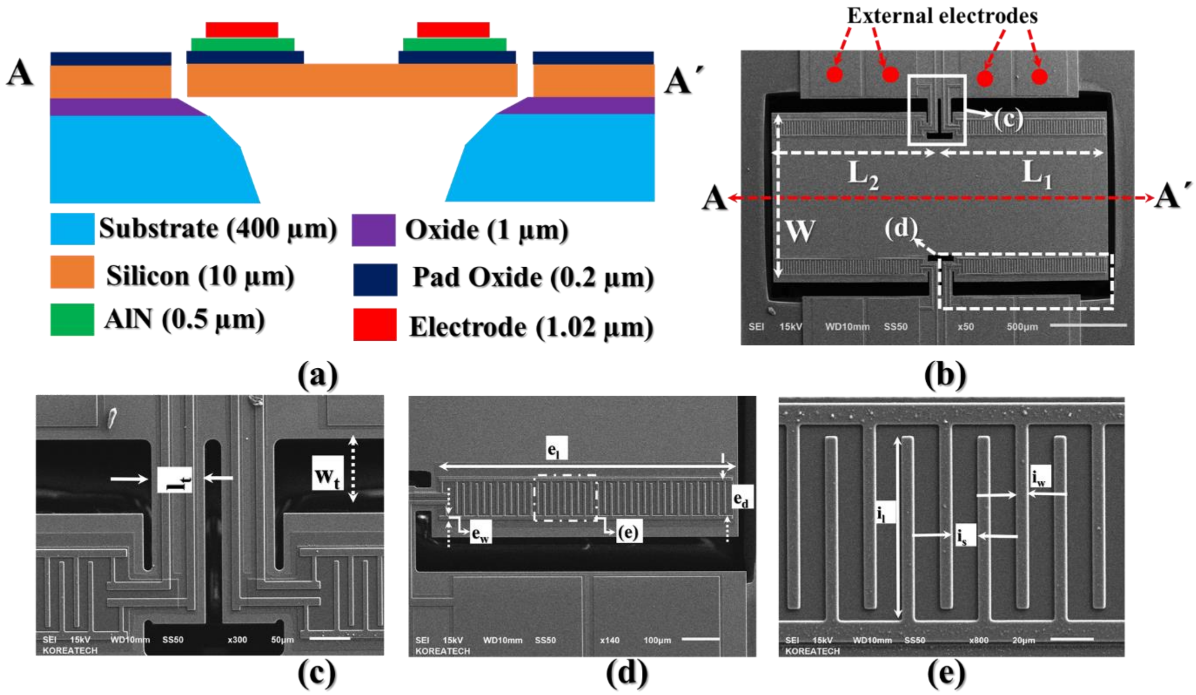

Fig. S1. Fabrication and device parameters. (a) cross-sectional view (not to scale) of the fabricated device (re-drawn from PiezoMUMPs manual **[SR_5]**), (b) SEM of the fabricated device where the boxes indicate the zoomed view of the torsional beam's structure, and D33 electrode's structure, (c) zoomed view of the torsional beam which was taken from a single side of the diaphragm, (d) zoomed view of the D33 electrode structure where the box indicates the zoom view of the interdigitated electrode's structure, and (e) zoomed view of the interdigitated electrodes.

Fig. S1(b) shows the scanning electron micrograph (SEM) of the fabricated device; where L_1, and L_2 the identical length of two diaphragms which are pivoted at the middle. Also, W is the width of the diaphragm, and external electrodes to connect with the measuring devices are mentioned in the bottom of the Fig. S1(b). Fig. S1(c) shows the zoomed view of the supportive torsional beam's structure which was used to anchored the diaphragms; where, l_t, and w_t are the length of each torsional beam, and width of each torsional beam, respectively. Note that, the incorporated torsional beams are identical, thus, only one pair of torsional beams was used to describe it. Fig. S1(d) shows the D33 electrode structure; where e_l, e_w, and e_s are the length of the main electrodes, width of the main electrodes, and gap of

the main electrodes, respectively. In-between the main electrode's gap, the interdigitated electrodes were patterned which is indicated using box in Fig. S1(d). Fig. S1(e) shows the zoomed view of the interdigitated electrodes of the fabricated device; where i_l, i_s, and i_w are presenting the length, spacing, and width of the interdigitated electrodes, respectively. It is noted that the value of all parameters which are discussed above are listed in Table S1.

3. Experimental setup:

The extended view of experimental setups which were shown in Figure 4(b) and Figure 6 in the **"main article"** is shown in Fig. S2. In Fig. S2(a), it can be noticed that the device under test (DUT) was mounted on a 1 m long rod and placed far from the measuring devices to avoid the to avoid the reflection from them. Before the placing the DUT, on top of the rod, the rotational stage (PRM1Z8, Thorlabs Inc.) was placed to get the directivity measurements. The top view along with the microscopic view of the developed device is shown in Fig. S2(b). Notably, the measurements shown in Figures 2(c)-(d) and Figures (a)-(f) (in the **"main article"**) were measured from 0.4 m. Moreover, the directionality measurements were carried out by rotating the rotation stage. The stage is controlled by a single channel motor controller (KDC101, Throlabs Inc.).

Further, the signal from each diaphragm was connected to a charge amplifier (SR570, Stanford Research Systems), and then each sensing signal was recorded using a lock-in amplifier (SR830, Stanford Research Systems). Whereas, the sound was generated using a function generator (DS345, Stanford Research Systems). The schematic view of until this point is shown in Figure 6 in the **"main article"**.

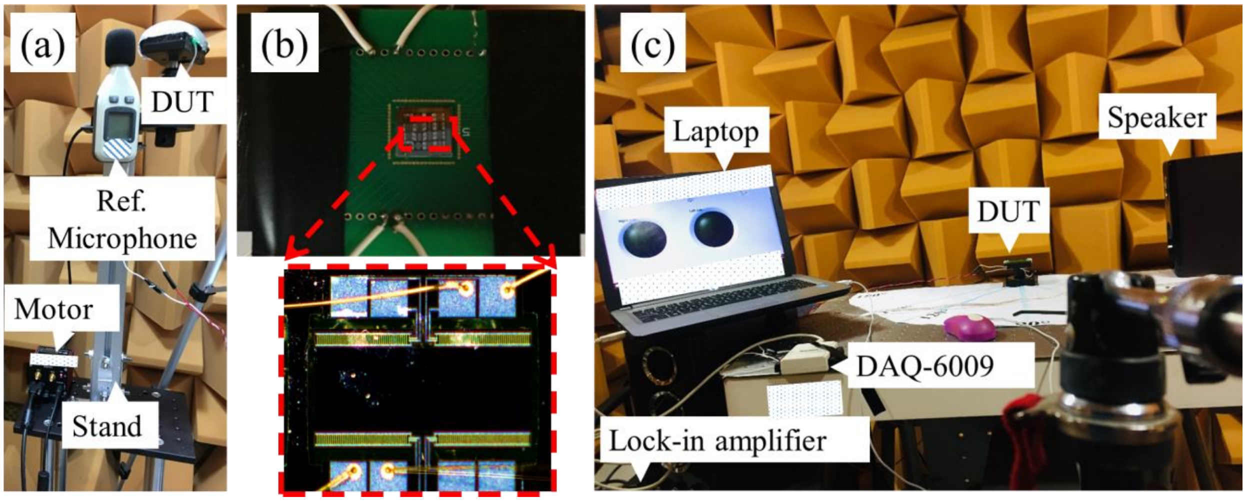

Fig. S2: Experimental setups in an anechoic chamber. (a) extension of Figure 6 (in the **"main article"**), (b) closed view of the developed device, and (c) extension of Figure 4(b) (in the **"main article"**).

Further, the experimental setup is shown in Fig. S2(c) was modified to demonstrate the SSL both in frequency domain and azimuth angle. To do that, the authors had to place to the rotational stage on a table in order to apply the sound source in different angles as shown in Fig. S2(c). Moreover, before placing the device, the table was calibrated to

control the reflections as much as possible. Then, the sensing signal was directly connected to a lock-in amplifier in order to check the credibility of the developed device without having the amplification. After that, the signal was connected to a data acquisition device (DAQ-6009, National Instruments) to interface with the LabVIEW 2015 version software. In the LabVIEW software, two logic were developed to demonstrate the SSL in frequency domain and azimuth angle simultaneously. Using Fig. S2(c), the SSL in frequency domain and azimuth angle were perfectly achieved which are shown in **"SV_2.mp4"**, and **"SV_3.mp4"**, respectively.

4. Noise calculation:

4.1. Noise model:

Fig. S3 shows the noise model of the developed bio-inspired piezoelectric MEMS directional microphone which was made followed by the foundry work on the piezoelectric MEMS directional microphone **[SR_2]**. Where, V_{off}, C_{eb}, n_{vs}, n_{is}, R_m, C_c, n_{if}, C_f, R_f, n_{vf}, n_i, G, and V_o are the off-sound response of the directional microphone, blocking capacitance, voltage noise of the sensor, current noise of the sensor, resistance followed by the material dielectric loss tangent, cable capacitance, current noise of the amplifier, feedback capacitor, feedback resistor, voltage noise of the amplifier, total input noise at the input terminal of the amplifier, amplifier's gain, and total predicted noise at the end of the amplifier, respectively.

The voltage noise due to the material can be defined as [**SR_2**],

$$\bar{n}_{vs}{}^{2} = 4K_bTR_m = 4K_bT \times \frac{\tan\delta}{\omega \times C_{eb}} \quad \left(\frac{V}{\sqrt{Hz}}\right) \tag{S1}$$

where, K_b, T, tanδ, and ω are the Boltzmann constant, room temperature, dielectric loss tangent, and frequency in radian/s, respectively.

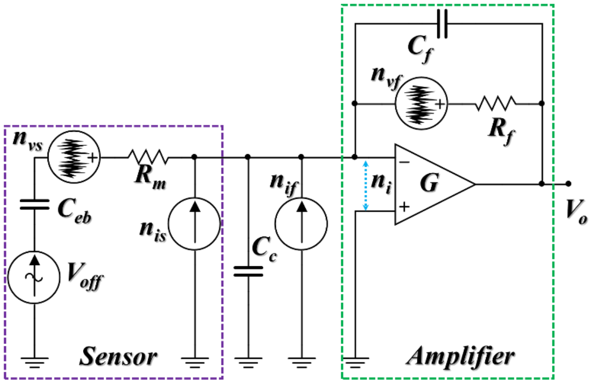

Fig. S3. Noise model of the developed work.

4.2. A-weighted noise:

The A-weighted broadband noise can be defined as [**SR_6**],

$$N_o = 10 \times \log_{10}\left(\sum_{i=1}^{n} 10^{\frac{N_i}{10}}\right) \quad (dBA) \tag{S2}$$

where, N_o, and N_i are the overall A-weighted noise, and A-weighted noise of each sensing frequency (i=1,2,3,…,n). The A-weighted noise of each sensing frequency can be derived as **[SR_7]**,

$$N_i = V_m - dBA(f) \tag{S3}$$

where, V_m is the measured voltage in ref. 1V, and dBA(f) is the frequency weighting.

5. References

[SR_1]. Rahaman, A., Ishfaque, A. & Kim, B. Effect of torsional beam length on acoustic functionalities of bio-inspired piezoelectric MEMS directional microphone. *IEEE Sensors J.* **19**, 6046-6055, DOI: https://doi.org/10.1109/JSEN.2019.2909501 (2019).

[SR_2]. Rahaman, A., Ishfaque, A., Jung, H. & Kim, B. Bio-inspired rectangular shaped piezoelectric MEMS directional microphone. *IEEE Sensors J.* **19**, 88–96, DOI: 10.1109/JSEN.2018.2873781 (2019).

[SR_3]. Kuntzman, Michael L., et al. "Micromachined in-plane pressure-gradient piezoelectric microphones." *IEEE Sensors Journal* **15**.3 (2014): 1347-1357.

[SR_4]. Kuntzman, M. L. & Hall, N. A. Sound source localization inspired by the ears of the *Ormia ochracea.Appl. Phys. Lett.***105**, 033701, DOI: 10.1063/1.4887370 (2014).

[SR_5]. Cowen, A., Hames, G., Glukh, K. & Hardy, B. PiezoMUMPs design handbook. MEMSCAP Inc1, DOI: http://www.memscapinc.com/__data/assets/pdf_file/0020/5915/PiezoMUMPs.DR.1.3a.pdf (2014).

[SR_6]. R. N. Miles, Analysis of acoustic signals, in: Physical Approach to Engineering Acoustics, Springer, 2019, pp. 1–31.doi:10.1007/978-3-030-22676-3.

[SR_7]. Mohamad, N., Iovenitti, P. & Vinay, T. Modelling and optimization of a spring-supported diaphragm capacitive MEMS microphone. Engineering, 2, 762–770, DOI: https://doi.org/10.4236/eng.2010.210098 (2010).
